# Being Praised for Prosocial Behaviors Longitudinally Reduces Depressive Symptoms in Early Adolescents: A Population-Based Cohort Study

**DOI:** 10.1192/j.eurpsy.2023.724

**Published:** 2023-07-19

**Authors:** D. Nagaoka, N. Tomoshige, S. Ando, M. Morita, T. Kiyono, S. Kanata, S. Fujikawa, K. Endo, S. Yamasaki, M. Fukuda, A. Nishida, M. Hiraiwa-Hasegawa, K. Kasai

**Affiliations:** ^1^The Department of Neuropsychiatry, The University of Tokyo; ^2^ Research Center for Social Science & Medicine, Tokyo Metropolitan Institute of Medical Science; ^3^The Department of Neuropsychiatry, Teikyo University School Medicine, Tokyo; ^4^Department of Psychiatry and Neuroscience, Gunma University Graduate School, Maebashi; ^5^School of Advanced Sciences, SOKENDAI (The Graduate University for Advanced Studies), Hayama, Japan

## Abstract

**Introduction:**

Depression is highly prevalent and causes a heavy burden in adolescent life. Being praised for prosocial behavior might be a preventive factor because both being praised and prosocial behavior are protective against depression. However, no study has investigated the association between experiences of being praised for prosocial behavior and depressive symptoms in adolescents.

**Objectives:**

Here, we investigated the longitudinal relationship between being praised for prosocial behavior and depressive symptoms in adolescents.

**Methods:**

In Tokyo Teen Cohort study (TTC), an ongoing prospective population-based cohort study, we collected 3,171 adolescents’ data on self-reported experiences of being praised for prosocial behavior, depressive symptoms, and caregiver-evaluated prosocial behavior. Ten-year-old children were asked to freely describe answers to the question “What are you praised for?”. Only children who clearly answered that they were praised for their prosocial behavior were designated the “prosocial praise group.” The degree of depression at ages 10 and 12 was measured with the Short Mood and Feelings Questionnaire (SMFQ), a self-report questionnaire about depression. Objective prosocial behavior of the 10 year-old children was assessed by the Strength and Difficulty Questionnaire (SDQ). Multiple linear regression analysis was performed using the SMFQ score at age 12 as the objective variable and being praised for prosocial behavior as the main explanatory variable, and the SMFQ score at age 10 and the objective prosocial behavior at age 10 were included as confounders.

**Results:**

3,007 pairs of child and their primary caregiver participated in the second data collection at the age of 12 years (follow-up rate was 94.8%). Regarding the question “What are you praised for?”, 845 (28.1%) children answered that they were praised for prosocial behavior. Depressive symptoms (SMFQ scores) in the “prosocial praise group” were significantly lower than those in the other group both at age 10 (4.3 ± 4.4 vs. 4.9 ± 4.6, *p* < 0.001) and at age 12 (3.4 ± 4.2 vs. 4.0 ± 4.6, *p* < 0.01). In the single regression analysis, the children who reported being praised for prosocial behavior at age 10 had significantly lower depressive symptoms at age 12 (partial regression variable: −0.57, 95% confidence interval (CI) [−0.96, −0.17]). This association remained significant after adjusting for confounders, including baseline depressive symptoms (partial regression variable: −0.44, 95% CI [−0.80, −0.08]). Prosocial behavior alone was not associated with depressive symptoms.

**Image:**

**Figure:**
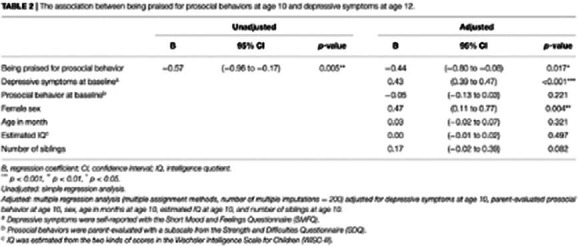

**Conclusions:**

Being praised for prosocial behavior rather than objective prosocial behavior at 10 years of age predicted lower depressive symptoms 2 years later. Praise for adolescents’ prosocial behavior can be encouraged to prevent depression.

**Disclosure of Interest:**

None Declared

